# One year of digital health applications (DiGA) in Germany – Rheumatologists’ perspectives

**DOI:** 10.3389/fmed.2022.1000668

**Published:** 2022-10-25

**Authors:** Jutta G. Richter, Gamal Chehab, Philipp Stachwitz, Julia Hagen, Denitza Larsen, Johannes Knitza, Matthias Schneider, Anna Voormann, Christof Specker

**Affiliations:** ^1^Policlinic for Rheumatology and Hiller Research Unit for Rheumatology, Medical Faculty, Heinrich-Heine-University Düsseldorf (HHUD), University Clinic, Düsseldorf, Germany; ^2^Health Innovation Hub of the Federal Ministry of Health (hih), Berlin, Germany; ^3^Department of Internal Medicine Rheumatology and Immunology, Friedrich-Alexander-University Erlangen-Nürnberg (FAU), Universitätsklinikum Erlangen, Erlangen, Germany; ^4^German Society for Rheumatology, Berlin, Germany; ^5^Department of Rheumatology and Clinical Immunology, KEM Kliniken Essen-Mitte, Essen, Germany

**Keywords:** DiGA, digital health, rheumatology, digital health applications, COVID-19

## Abstract

**Background:**

Based on given legislation the German approach to digital health applications (DiGA) allows reimbursed prescription of approved therapeutic software products since October 2020. For the first time, we evaluated DiGA-related acceptance, usage, and level of knowledge among members of the German Society for Rheumatology (DGRh) 1 year after its legal implementation.

**Materials and methods:**

An anonymous cross-sectional online survey, initially designed by the health innovation hub (think tank and sparring partner of the German Federal Ministry of Health) and the German Pain Society was adapted to the field of rheumatology. The survey was promoted by DGRh newsletters and Twitter-posts. Ethical approval was obtained.

**Results:**

In total, 75 valid response-sets. 80% reported to care ≥ 70% of their working time for patients with rheumatic diseases. Most were working in outpatient clinics/offices (54%) and older than 40 years (84%). Gender distribution was balanced (50%). 70% knew the possibility to prescribe DiGA. Most were informed of this for the first time via trade press (63%), and only 8% via the scientific/professional society. 46% expect information on DiGA from the scientific societies/medical chambers (35%) but rarely from the manufacturer (10%) and the responsible ministry (4%). Respondents would like to be informed about DiGA via continuing education events (face-to-face 76%, online 84%), trade press (86%), and manufacturers′ test-accounts (64%). Only 7% have already prescribed a DiGA, 46% planned to do so, and 47% did not intend DiGA prescriptions. Relevant aspects for prescription are provided. 86% believe that using DiGA/medical apps would at least partially be feasible and understandable to their patients. 83% thought that data collected by the patients using DiGA or other digital solutions could at least partially influence health care positively. 51% appreciated to get DiGA data directly into their patient documentation system/electronic health record (EHR) and 29% into patient-owned EHR.

**Conclusion:**

Digital health applications awareness was high whereas prescription rate was low. Mostly, physician-desired aspects for DiGA prescriptions were proven efficacy and efficiency for physicians and patients, risk of adverse effects and health care costs were less important. Evaluation of patients’ barriers and needs is warranted. Our results might contribute to the implementation and dissemination of DiGA.

## Introduction

Digital medicine is a great challenge for rheumatology as for other medical disciplines that is being encouraged by various, very different developments such as the digitalization of existing processes, digital health applications, but also methods of artificial intelligence (1). Digital healthcare concepts have already entered Rheumatology encompassing real-time, direct communication (e.g., via video consultations) and asynchronous exchanges of information including remote-patient monitoring as well as patient identification and stratification (e.g., via email, ICT platforms, Apps, and wearables) (2–5). Within the COVID-19 pandemic telemedicine tools rapidly and widely gained more acceptance as indispensable management tools for continuous care in rheumatic diseases and have been adopted in position papers and guidelines for the management of rheumatic diseases in adult patients (6–8).

Digital health applications (Digitale Gesundheitsanwendungen, DiGA) represent a novel digital healthcare concept established on given German legislation (§§33a and 139e SGB V, Social Code Book V). DiGA are medical devices of a low-risk category (Class I or IIa according to the Medical Device Regulation (EU) 2017/745) whose main function relies on digital technologies. They are intended to be used by patients in the detection, monitoring, treatment, or mitigation of disease or the detection, treatment, mitigation, or compensation for injury or disability (§§33a SGB V). Since October 6th, 2020, such reimbursable therapeutic software products approved by the Federal Institute for Drugs and Medical Devices (BfArM) can be prescribed by physicians and psychotherapists. DiGA and the corresponding relevant prescription information (e.g., indications and contraindications) are listed transparently in the DiGA directory (9).^1^ They may support physicians of all disciplines and patients in the diagnostics and treatment in the future (9).

The health innovation hub (hih), a think tank and sparring partner of the German Federal Ministry of Health, and the German Pain Society (Deutsche Schmerzgesellschaft) initially designed the questionnaire in order to assess opinions, experiences, and assessments of physicians on the topics of opportunities, risks, and future value of medical applications and DiGA in particular.

Based on this tool we evaluated for the first time the DiGA-related acceptance, usage, and level of knowledge among members of the German Society for Rheumatology (DGRh) 1 year after its legal implementation.

## Materials and methods

From 10th November 2021 until 15th December 2021 an anonymous, voluntary, cross-sectional nationwide online survey was conducted among members of the German Society for Rheumatology (DGRh).

The applied questionnaire was adapted for rheumatological aspects to the questionnaire that was initially developed by the health innovation hub (hih) and the German Pain Society and applied to members of the German Pain Society in 2021 (10). First, general questions were asked on the topic of digital medicine. Further questions dealt with the topic of DiGA. Furthermore, the questionnaire included the validated questionnaire for the assessment of affinity for technology. The instrument comprises 19 items scored on 1–5 Likert Scales covering four subscales: Enthusiasm for technology, competence in dealing with technology, positive consequences of its use, and negative consequences of technology (11).

The Checklist for Reporting Results of Internet E-Surveys (CHERRIES) was followed (12). A questionnaire containing 68 questions using pre-given answering options (including free-text options where necessary) or Likert scales (five possible answers) to indicate positive-to-negative statements was applied. Adaptive questioning was implemented. Due to content-related reasons items were not randomized or alternated. For two questions response options were alternated. It was not possible to leave questions unanswered except for the indication of personal data.

The survey was promoted via newsletters sent out to DGRh newsletter recipients, a Twitter post and an E-Mail sent out to the “German Regional Cooperative Rheumatology Centers”. The newsletters of the DGRh were sent out to *n* = 1,669 subscribers. The target group of newsletter recipients is predominantly male (58%) and more than 81% are older than 40 years (personal communication, data on file at DGRh). Incentives were not offered.

We obtained ethical approval from the local ethic committee (local study number 2021-1737). The study was registered to the German Clinical Trials Register (Identifier DRKS00027939) retrospectively.

Data collection was performed using the survey tool LimeSurvey.^2^ Data were extracted from the survey tool as MS-Excel and CSV files and imported to IBM SPSS to perform statistical analyses (IBM SPSS Statistics version 27). Predominantly descriptive statistics were executed. Values are expressed as valid percentages for discrete variables, or as mean ± standard deviation (SD) for continuous variables. Age subgroups were dichotomized into two groups: up to 39 years of age and above 40 years of age. Differences of distribution were tested via Chi Square and - where appropriate - non-parametrically (Mann–Whitney *U* test and Kruskal Wallis Tests). All statistical tests were performed two-tailed, *p*-values less than 0.05 were considered significant.

## Results

### Cohort characteristics

In the log files of the survey tool *n* = 96 visitors were registered. Eight only logged into the survey but did not answer any question and *n* = 13 stated that they do not take care of patients. Thus, these were excluded from the analysis and we report on *n* = 75 valid data sets. Of these 80% (*n* = 59/74) reported that they care ≥ 70% of their working time for patients with rheumatic diseases. Most were working in outpatient clinics and offices (54%, *n* = 38/70) and older than 40 years of age (84%, *n* = 56/70). Gender distribution was balanced (50%).

Ninety-three (*n* = 53/57) percent of the participants stated that they were well informed on the topic of digital medicine and 95% (*n* = 59/62) reported that they actively inform themselves on the topic of digital medicine. In these regards, no statistical differences were noted for gender and age subgroups.

According to the validated “questionnaire for recording affinity for technology” the respondents’ enthusiasm for technology scored to 2.9 ± 0.8 (mean ± SD), the competence in dealing with technology to 3.4 ± 0.8 (mean ± SD), the positive consequences of technology to 3.3 ± 0.6 (mean ± SD), and the negative consequences of technology to 3.1 ± 0.7 (mean ± SD). No significant differences were observed between females and males. The competence in dealing with technology, the positive consequences of technology, and the negative consequences of technology scored significantly higher in those aged below 40 years of age (all *p* < 0.05) while enthusiasm for technology was similar.

### DiGA related acceptance and knowledge

Seventy percent (*n* = 49/70) were aware of the possibility to prescribe DiGAs, no gender or age difference was notable. Most were informed of this for the first time via trade press (63%, *n* = 31/49), and only 8% (*n* = 4/49) via the professional society. Fourty six percent (*n* = 32/70) of the participants expected information on DiGAs to mostly come from trusted bodies like the professional societies (46%, *n* = 32/70) and the medical chambers (36%, *n* = 25/70) but rarely from the DiGA manufacturer (10%, *n* = 7/70) and the responsible ministry (4%, *n* = 3/70). Distribution was similar for age and gender groups.

Respondents would like to be informed about DiGAS via continuing education events [face-to-face (76%, *n* = 53/70) or online (84%, *n* = 59/70)], the trade press (86%, *n* = 60/70), and also via manufacturers test accounts (64%, *n* = 45/70). No significant differences were noted for age groups. Females preferred all information resources significantly more often than males.

Overall, 38% (*n* = 27/71) reported a positive attitude toward DiGA/medical apps, and 57% (*n* = 41/72) knew dedicated DiGA/medical apps.

### DiGA usage

**FIGURE 1:**
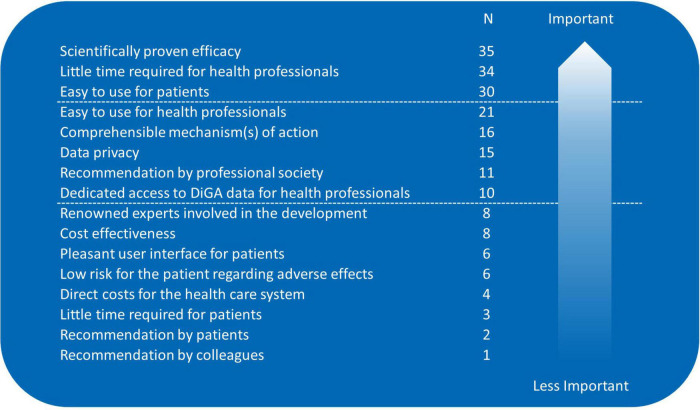
Aspects relevant for digital health application (DiGA) prescriptions; sorted by importance/number of mentions (participants needed to pick their three most relevant aspects from a pre-given list).

Only 7% (*n* = 5/70) of the respondents have already prescribed a DiGA, 46% (*n* = 32/70) planned to do so, and 47% (*n* = 33/70) do not intend DiGA prescriptions. Distribution was significantly different between male and female participants: more females planned the prescription (*p* = 0.041). Distribution was similar for age groups. Up to three most relevant aspects for prescription could be picked from a pre-given list, data are listed in Figure 1.

### Usage of digital health applications/medical apps in routine rheumatology care

Most (86%, *n* = 60/70) believe that using DiGA/medical apps would at least partially be feasible and understandable to their patients. The majority of respondents (83%, *n* = 58/70) thought that data collected by the patients using DiGA or other digital solutions can at least partially influence health care positively.

Half of the respondents (51%, *n* = 36/70) appreciated getting digitally collected patient data directly into their patient documentation system resp. clinical electronic health record (EHR) and 29% (*n* = 20/70) favor a transport of the data into patient-owned EHRs. All these four views were distributed similarly for age and gender groups.

**FIGURE 2:**
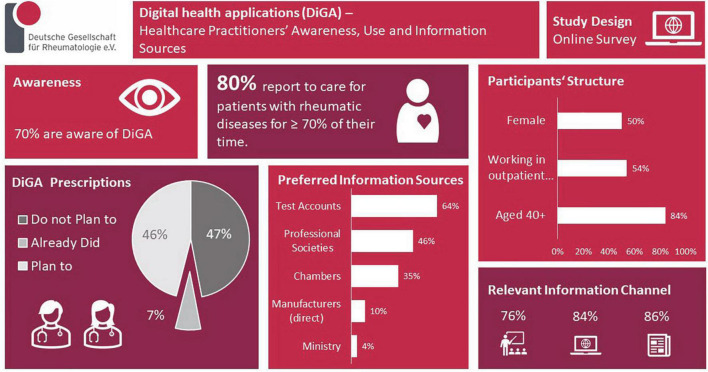
Infographic summarizing the study results.

Results are summarized in the infographic (Figure 2).

## Discussion

To our knowledge, this is the first study that evaluated DiGA-related acceptance, usage, and level of knowledge among rheumatologists pointing out currently low usage, but high acceptance among rheumatologists. Multiple barriers could be identified to guide and facilitate further DiGA implementation into clinical routine. The results enable manufacturers, as well as medical societies, to meet the demands and expectations for the implementation of DiGA for rheumatic diseases and its co-morbidities or habits (e.g., smoking, physical activities) placed on them. Since the BfArM DiGA directory did not yet list a rheumatology specific DiGA at the time of the survey, our data can be considered relatively unbiased.

We regarded the respondents according to sociodemograpic data and time spent for clinical work on patients with rheumatic diseases as experienced, clinically active rheumatologists. As gender distribution among respondents was balanced, we assume to have avoided a gender bias.

The respondents considered themselves familiar with digital medicine. Respondents’ recordings of “affinity for technology” demonstrate similar data compared to those obtained via the German Pain Society (personal communication, data on file) and the German Respiratory Society (13).

Manufacturers test accounts were appreciated by nearly two thirds (64%) of the respondents. This is in line with a recently published review that welcomes unlimited test access to DiGA, e.g., for demonstration purposes during the required educational conversations between physicians and the patients (9). According to Haserück “limited test access is therefore available ‘at any time upon request from the companies’ for physicians and psychotherapists” (14).

Thirty-eight percent of our respondents reported a positive attitude toward DiGAs/medical apps. This number is lower than the number published from a large German survey where 62% of the participants (97% from outpatient clinics) “viewed the fact that physicians can prescribe DiGA as positive or very positive” (15). An online-survey focusing on the impact of the COVID-19 pandemic on usage, preferences and perception of digital health application reported that 76% of German rheumatologists believed that digital health applications (DHA) are useful in the management of rheumatic and musculoskeletal diseases, and 71% of the rheumatologists indicated that their attitude toward DHA had changed positively (16). This is also reflected by the reported increasing usage of medical apps among German rheumatologists (17).

A low number of our participants already prescribed DiGA (7%) but 46% planned to do so. This number is quite similar to the members of the German Respiratory Society where 47.2% had prescribed or planned to prescribe DiGA (13). However, our number is higher than the number from the above-mentioned German survey from October 2020 where 30% (*n* = 393/1299) planned to prescribe DiGA (15). In that study recommendations from medical associations (80%) and medical colleagues (79%) were seen as the most impactful remedies to support professionals who are unsure of prescribing DiGA (15). In our cohort the number of respondents asking for information on DiGAs from trusted bodies such as the professional/scientific societies and the medical chambers was lower (46% respectively 36%). Medical colleagues’ recommendations were of minor importance. Efficacy and efficiency were the most physician-desired aspects for DiGA prescriptions in our cohort. This is consistent with data from Priebe et al. who report that more than 70% of healthcare professionals place particular emphasis on a mechanism of action validated in controlled clinical trials as well as Dahlhausen et al. reporting that 55% of the healthcare professionals perceived insufficient evidence as a barrier to prescribing DiGA (10, 15).

Most respondents felt DiGA or medical app use would at least partially be feasible and understandable to their patients (86%), and digital data collection by patients (e.g., via DiGA) can at least partially influence health care positively (86%). Thus, we anticipate that use of DiGA will be valued in future routine modern patient management.

This is supported by the fact that half of the respondents appreciated getting data collected digitally from the patient directly into their patient documentation system resp. EHR reflecting interest in data from digital applications for the caring situation. This is in line with results from 2019 showing that 26% of German rheumatologists already used electronic patient-reported outcomes (ePRO), and 44% were planning to switch to ePRO. The most commonly cited barrier was the unawareness of suitable software solutions (18). It may also reflect the reported expectation that DiGA use will give the physician more time to interact with the patient and also provide additional information that may be used to optimize individual therapy (9). Interoperability of DiGA has already been recognized as an important aspect (19) and recent German legislation has been adopted to ensure transfer to the German electronic patient record (20).

Although we expected data security issues to be of relevance for the implementation of DiGA this issue was of minor relevance in our cohort. An explanation could be that respondents expect only General Data Protection Regulation (GDPR) compliant, CE marked DiGA in the directory since these are requirements that have to be met in order to be listed (9, 21).

However, as there is hardly any experience with liability issues in the DiGA context (9) their implementation might still be limited. Moreover, the current legal regulations are not yet seen as final and proposals for adaptations have been made (20). The ongoing developments regarding the creation of the European data space might influence the market as well (22).

DiGA prescriptions could be hampered in clinical practice by the need of a documented educational conversation in advance of the prescription (9). Questions that ascend when patients use the DiGA will predominately be asked to the prescribing physician and unforeseen side effects are not yet systematically recorded (9). These DiGA related tasks require resources probably not yet foreseen by the respondents asking for educational training on DiGA-related processes to provide information “on the current and upcoming challenges that the various stakeholders face on the way to integrate DiGA into standard care in a widespread and sustainable way” (23). The necessity to provide physicians with education for more expertise and competence regarding digitalization in the DiGA context has also been identified by others (24). Interestingly, in our cohort, these trainings on DiGA were expected to be carried out by the professional societies and rarely from the manufacturers. This is somewhat different to known information resources for updates on medical knowledge [e.g., on (new) medications] as in a study from Kosteniuk et al. 46% valued pharmaceutical sales representatives and 38% of colleagues as information resources (25). Respondents’ interest in educational events about DiGA (76%) corresponds to recently reported physicians’ interest in medical congresses/educational events for updates on medicinal products (26). Our female respondents indicated interest in various formats of education (face-to-face, online, etc.) more often, it remains open whether a gender-specific education is necessary although knowledge on digital medicine was comparable to males.

### Limitations

Compared to the number of DGRh members our cohort is quite small. This was attributed to the fact that during the COVID pandemic physicians were facing a multitude of online surveys. In addition, due to hih closing at the end of 2021 parallel to the end of the 19th legislative period of the German Bundestag in October 2021 the survey could not be extended. Our participants were digital affine. Thus, a selection and non-response bias cannot be excluded. The applied questionnaire, which was initially developed in cooperation between hih and the German Pain Society, has not yet been validated except for the part of the validated questionnaire for the assessment of affinity for technology. We did not include patients’ experiences on DiGA and their preferences.

## Conclusion

Our results indicate that DiGA awareness was already high whereas prescription rate was low among German rheumatologists. Mostly, physician-desired aspects for DiGA prescriptions were proven efficacy and efficiency for physicians and patients, risk of adverse effects and health care costs were less important. Evaluation of patients’ barriers and needs are warranted. Our results might contribute to the implementation and dissemination of DiGA.

## Data availability statement

The data supporting the conclusions of this article are available on reasonable request by the authors.

## Ethics statement

The studies involving human participants were reviewed and approved by Ethic committee of the Medical Faculty, Heinrich-Heine-University Düsseldorf. Written informed consent for participation was not required for this study in accordance with the national legislation and the institutional requirements.

## Author contributions

JR, PS, DL, JH, GC, and CS designed, performed, analyzed the study, drafted the manuscript, and analyzed the data. JK, MS, and AV designed and performed the study. All authors contributed to the manuscript revision, read, and approved the submitted version.

## Footnotes

1
https://diga.bfarm.de/de/verzeichnis

2
https://www.limesurvey.org
